# Immunosuppressive tumor microenvironment and advance in immunotherapy in melanoma bone metastasis

**DOI:** 10.3389/fimmu.2025.1608215

**Published:** 2025-08-27

**Authors:** Yiqun Ma, Lin Zhang, Weimin Liu

**Affiliations:** ^1^ Department of Burns and Plastic Surgery, Kunming Children′s Hospital, Children′s Hospital Affiliated to Kunming Medical University, Kunming, China; ^2^ Department of Orthopaedic, The Third Affiliated Hospital of Shenzhen University (Luohu People’s Hospital), Shenzhen, China; ^3^ Department of Dermatology, The Affiliated Hospital of Yunnan University, Kunming, China

**Keywords:** melanoma, bone metastasis, osteoclasts, tumor microenvironment, immune checkpoint inhibitors, immunotherapy

## Abstract

Melanoma frequently develops bone metastases, leading to skeletal-related events and poor survival. The tumor microenvironment (TME) plays a pivotal role in melanoma progression, bone metastasis, and immunotherapy resistance. Key immunosuppressive cells including myeloid-derived suppressor cells (MDSCs), tumor-associated macrophages (TAMs), regulatory T cells (Tregs), and cancer-associated fibroblasts (CAFs) promote immune evasion and osteolytic bone destruction via RANKL-dependent and -independent mechanisms. Immune checkpoint inhibitors (ICIs), including anti-CTLA-4 and anti-PD-1/PD-L1 therapies, have revolutionized melanoma treatment, yet resistance remains common due to TME immunosuppression. Emerging strategies, such as combination therapies, aim to enhance efficacy by reshaping the TME. This review synthesizes current knowledge on TME-driven immunosuppression, bone metastasis mechanisms, and immunotherapeutic advancements, offering insights into overcoming resistance and improving patient outcomes.

## 1 Introduction

Melanoma is an aggressive skin cancer characterized by early metastasis and accounts for nearly 90% of deaths from malignant skin tumors despite its relatively low incidence (1, 2). While early-stage cases are surgically curable with favorable outcomes, advanced melanoma exhibits high invasiveness, poor response to radiotherapy and chemotherapy, and a five-year survival rate of only 30% (3, 4). Up to 17% of patients develop metastatic bone disease, leading to skeletal-related events, reduced quality of life, and poorer survival (5). Bone metastasis depends on interactions between tumor cells and the tumor microenvironment (TME), particularly immune components (6, 7).

Recent progress in immunotherapy has offered promising treatment strategies for melanoma (8, 9). Its high immunogenicity enables the immune system to recognize tumor-associated antigens, facilitating immune checkpoint blockade (10). Agents targeting immune checkpoint inhibitors (ICIs), including CTLA-4, PD-1, and PD-L1, have significantly improved survival and are now the standard of care for advanced melanoma (11). However, the immunosuppressive nature of the TME remains a critical barrier, limiting treatment responses and contributing to resistance in many patients (12). Consequently, understanding the mechanisms of immunotherapy and the composition and function of the TME is critical for effectively controlling melanoma progression and improving overall patient survival (13). This review focuses on how the TME shapes melanoma development, bone metastasis, and immunotherapy response, integrating current therapeutic approaches, analyzing their mechanisms and limitations, and providing insight into novel strategies for improving immunotherapy response in melanoma.

## 2 Immunosuppressive cells in tumor microenvironment of melanoma

### 2.1 Myeloid-derived suppressor cells

MDSCs represent a heterogeneous population of immature myeloid cells that exert immunosuppressive functions (14). During tumor progression, MDSCs are recruited and activated by multiple proinflammatory cytokines, such as prostaglandin E2 (PGE2), granulocyte colony-stimulating factor (G-CSF), granulocyte-macrophage colony-stimulating factor (GM-CSF), and CCR5, which are released within the TME (15). In melanoma, MDSCs produce immunosuppressive molecules including nitric oxide synthase, reactive oxygen species, and arginase-1, thereby inhibiting T-cell activation and inducing T-cell apoptosis and cell-cycle arrest (16). They also activate the STAT3 pathway, promoting epithelial–mesenchymal transition and expediting tumor immune escape (16). In addition to their immunomodulatory functions, MDSCs exert tumor-promoting effects through mechanisms independent of immune regulation, including stimulation of angiogenesis and establishment of a premetastatic niche (16). Clinically, an increased abundance of MDSCs within the tumor microenvironment has been correlated with poor responsiveness to immune checkpoint blockade in patients with melanoma (17). Accordingly, targeting MDSCs hold potential to improve treatment outcomes. Blattner et al. (18) showed that a CCR5-Ig fusion protein blocked CCR5–CCL5 interactions, inhibiting MDSC recruitment and prolonging survival in patients with melanoma. STAT3 inhibitor napabucasin induced MDSC apoptosis and extended survival in a murine melanoma model (16). These findings underscore the value of targeting MDSCs in melanoma therapy.

### 2.2 Tumor-associated macrophages

TAMs constitute a major component of the TME and can be broadly classified into M1 and M2 phenotypes based on their function and activation states (19). During melanoma progression, the recruitment of M2 macrophages exceeds that of M1 macrophages. M1-type TAMs secrete classical inflammatory cytokines that induce tumor cell necrosis, promote immune-cell infiltration into the TME, and eliminate tumor cells through phagocytosis and destruction (20), whereas M2-type TAMs exhibit immunosuppressive properties, facilitating tumor progression and distant metastasis through multifaceted mechanisms. First, they enhance tumor cell proliferation and invasion by secreting cytokines such as tumor necrosis factor beta (TNF-β), cyclooxygenase-2 (COX-2), and interleukin-10 (IL-10), along with matrix metalloproteinases (MMPs) that degrade the extracellular matrix and facilitate melanoma cell dissemination (20). Second, they promote angiogenesis by modulating adrenomedullin secretion, hypoxia-inducible factor-1α, and vascular endothelial growth factor (VEGF)-A (21). Third, they contribute to immune evasion by recruiting regulatory T cells (Tregs) and secreting immunosuppressive molecules such as IL-10, indoleamine 2,3-dioxygenase (IDO), and PD-L1 expression, which collectively suppress effector T cell (Teff) activity (21). Besides, they mediate resistance to targeted therapies via TNF-α-induced activation of the nuclear factor-κB pathway and upregulation of Sox family transcription factors in BRAF/MEK inhibitor-resistant melanoma models (22). Collectively, M2-type TAMs drive melanoma progression via the secretion of a broad range of bioactive mediators, making their selective inhibition or reprogramming toward the antitumor M1 phenotype an attractive therapeutic strategy. Han et al. (20) demonstrated that a baicalin-loaded nanocomplex targeting M2-type TAMs effectively inhibited melanoma growth by inducing proinflammatory M1 polarization and reshaping the TME, underscoring the therapeutic potential of TAM phenotype modulation.

### 2.3 Tregs and CD8^+^ T cells

Tregs contribute to immunosuppression and weak responsiveness to ICIs. They achieve immunosuppression by secreting inhibitory cytokines such as IL-10, IL-35, and TGF-β, as well as perforin and granzyme, which hamper the activation and proliferation of Teff and neutrophils (12, 23). Tregs also express multiple inhibitory checkpoint receptors, including lymphocyte-activation gene 3 (LAG-3), PD-1, and CTLA-4, thereby promoting immune tolerance (24). Hence, depleting Tregs within the TME has emerged as a promising therapeutic approach. Studies have shown that targeting Tregs in the TME restores Teff function and bolsters antitumor immunity in mouse models of melanoma (23). Notably, the intratumoral Teff/Treg ratio has been proposed as a predictive biomarker for immunotherapy outcomes (20). Cytotoxic CD8^+^ T cells recognize tumor antigens via MHC-I molecules and eliminate malignant cells through perforin and granzyme B-mediated apoptosis. They also secrete IFN-γ and TNF, which sustain antigen presentation and amplify T-cell responses (25). However, the immunosuppressive milieu of the TME often impairs CD8^+^ T-cell activity, facilitating tumor immune evasion.

### 2.4 NK cells and dendritic cells

NK cells can recognize melanoma cells that are resistant to T cell–mediated cytotoxicity and thus play an auxiliary role in anti-cancer immunity. Conversely, melanoma cells can suppress NK-cell function to evade immune surveillance. Studies have shown that melanoma cells secrete IDO and PGE2 to downregulate the expression of activating receptors (NKp30, NKp44, and NKG2D) on NK cells, thereby impairing their tumor-killing activity (26). Furthermore, Lee et al. (27) demonstrated that tumor cells can bind immunosuppressive receptors on the NK-cell surface to inhibit NK-cell activation. Consequently, promoting NK-cell infiltration and activation, via binding to tumor-cell surface ligands, targeting NK-cell–activating receptors, or blocking inhibitory receptors on NK cells, can enhance NK-cell–mediated tumor immunity and immune surveillance. Dendritic cells (DCs), the most potent antigen-presenting cells, orchestrate antitumor immunity through efficient cross-presentation and T cell priming. In melanoma, mature DCs expressing CD80 and CD86 are essential for activating tumor-specific T cells (28). However, melanoma cells within the TME secrete IL-6, IL-10, VEGF, and TGF-β, which disrupt DC recruitment and maturation, thereby impairing T-cell activation and promoting melanoma progression (28). However, tumor-derived cytokines and growth factors, notably IL-10, VEGF, and TGF-β, can skew DC differentiation toward a tolerogenic phenotype characterized by reduced expression of costimulatory molecules, impaired antigen presentation, and increased secretion of immunosuppressive cytokines (29–31). These tolerogenic DCs suppress effector T cell activation, promote the expansion of regulatory T cells, and contribute to immune evasion. Prokopi et al. (32) observed a significant reduction in DCs in human primary melanoma lesions, which was associated with poorer prognosis. A study by Tucci et al. (33) showed that metastatic melanoma patients have lower DC counts than non-metastatic patients; the number of DCs was negatively correlated with Treg count and positively correlated with low melanoma recurrence risk. Hence, enhancing DC activity in the TME is an effective therapeutic approach. Prokopi et al. (32) further developed a DC-boosting therapy that increases both the quantity and activation status of intratumoral DCs and Teff cells, thereby augmenting tumor immunogenicity and sensitizing melanoma to immunotherapy.

### 2.5 Cancer-associated fibroblasts

CAFs are the most abundant stromal cells in the TME of cutaneous malignant melanoma. They are highly heterogeneous and plastic, and can influence melanoma initiation, progression, metastasis, and drug resistance in various ways. First, CAFs secrete cytokines that favor melanoma invasion, including IL-6, IL-8, transforming TGF-β, β-catenin, fibroblast growth factor-2 (FGF-2), and VEGF (34). Second, CAFs suppress CD8^+^ T cells and NK cells. Érsek et al. (12) reported that CAFs inhibited CD8^+^ T cell cytotoxicity by depleting L-arginine. Romano et al. (34) further showed that CAF-derived matrix metalloproteinases and prostaglandin E2 reduced expression of activation receptors on NK cells, resulting in NK-cell inactivation. Third, CAFs promote resistance to immunotherapy and targeted therapy. Zhao et al. (35) discovered that CAFs secreted MMP9, which cleaved PD-L1 on the surface of melanoma cells and contributed to diminished responses to anti–PD-1 therapy. Diazzi et al. (36) found that CAFs produced neuregulin 1, along with large amounts of collagen and fibronectin, rendering melanoma cells unresponsive to MAPK inhibitors. Targeting CAFs may therefore offer a novel therapeutic strategy by improving antitumor immunity and immune surveillance in melanoma. Indeed, inhibiting MMP9/TGF-β expression reversed CAF-induced resistance to anti–PD-1 therapy and increased the ratio of CD8^+^ T cells to Tregs *in vivo* (35).

## 3 The role of immune cells in melanoma bone metastasis

Importantly, beyond their role in immune evasion, immunosuppressive cells such as MDSCs and TAMs also actively shape the metastatic niche in bone (37, 38). These cells secrete pro-osteoclastogenic cytokines (IL-6, TNF-α) and growth factors that promote osteoclast differentiation, thereby facilitating bone resorption (39, 40). Specifically, M2-polarized TAMs drive osteoclastogenesis via converging mechanisms. They secrete RANKL, M-CSF, IL-6 and TNF-α, which directly induce the differentiation and activation of osteoclast precursors. Concurrently, the release of matrix metalloproteinases and VEGF facilitates bone matrix remodeling and generates permissive niches for osteoclast function. Moreover, by shaping a cytokine-rich microenvironment, these TAMs sustain osteoclastic activity and promote persistent bone resorption (41, 42). TAMs, particularly the M2 phenotype, accumulate in the bone microenvironment where they enhance osteoclast activation (43, 44), while MDSCs serve as osteoclast precursors that, under the influence of RANKL and inflammatory cytokines, differentiate into mature osteoclasts and secrete IL-1β and cathepsin K, further amplifying bone resorption (45, 46). This dual role in immune suppression and skeletal remodeling establishes a permissive microenvironment for melanoma bone metastasis. Melanoma cells secrete various factors that induce TAMs recruitment, including VEGF-C, GM-CSF, M-CSF, and MCP-1 (47). Tumor-associated macrophages represent the predominant inflammatory cell population within both primary and metastatic melanoma lesions.

### 3.1 RANKL-dependent osteoclast formation

Clinical studies have demonstrated a significant correlation between increased TAM infiltration and enhanced melanoma aggressiveness (48). Functionally, these TAMs secrete a variety of pro-tumorigenic mediators such as IL-8, VEGF, and fibroblast growth factor (FGF), which collectively contribute to tumor progression and neovascularization in melanoma (49–51). Metastatic melanoma lesions are primarily osteolytic, driven by osteoclasts rather than tumor cells themselves (52, 53). Osteoclasts originate from hematopoietic mononuclear progenitor cells and belong to the mononuclear phagocyte system (54). These osteoclast precursor cells circulate among monocytes and exhibit characteristic monocyte/macrophage surface markers (52, 55). Their differentiation process is regulated by two essential factors: M-CSF and the RANK-RANKL signaling pathway. Osteoclast precursors expressing RANK interact with RANKL-presenting cells in bone tissue, while this interaction can be negatively regulated by osteoprotegerin secreted by osteoblasts and other cell types (56, 57). TAMs in melanoma metastases express CD14 but lack osteoclast markers (TRAP, VNR) and resorptive capacity until exposed to RANKL and M-CSF, inducing TRAP^+^ VNR^+^ multinucleated osteoclast formation. Melanoma-stromal interactions critically influence tumor progression and metastasis. Melanoma-associated fibroblasts may promote osteoclast formation and activation via soluble RANKL, akin to fibroblasts in giant cell tumors of bone (58), unlike normal fibroblasts from skin or bone marrow stroma (59–61). Tumor-associated stromal cells in primary melanoma not only promote cancer progression but also potentially contribute to bone metastasis by inducing osteoclast differentiation and subsequent bone destruction (62). The precise mechanisms underlying osteolytic lesions in melanoma remain unclear but involve RANKL-dependent crosstalk between tumor cells, stromal components, and osteoclast precursors.

### 3.2 RANKL-independent osteoclast formation

Emerging evidence suggests that osteoclastogenesis can be activated through RANKL-independent mechanisms mediated by various cytokines and growth factors, such as TNF-α, IL-6, IL-8, and TGF-β, which promote the differentiation of both bone marrow-derived and circulating osteoclast precursors (63–65). Notably, these cytokine-induced osteoclasts exhibit distinct morphological and functional differences compared to their RANKL-stimulated counterparts. While RANKL stimulation typically produces large multinucleated osteoclasts capable of extensive lacunar resorption, exposure to TNF-α and IL-1 results in the formation of significantly smaller osteoclasts containing fewer than four nuclei (48). These cytokine-derived osteoclasts demonstrate limited resorptive capacity, typically creating only single resorption pits, reflecting their alternative differentiation pathway. In the melanoma TME, these pro-osteoclastogenic cytokines activate intracellular signaling pathways that further amplify bone destruction and immune suppression (6). TNF-α engages TNF receptor 1/2, leading to activation of the NF-κB pathway through the IκB kinase (IKK) complex, which induces nuclear translocation of NF-κB subunits and transcription of osteoclastogenic and inflammatory genes (66, 67). IL-6 signals primarily via the gp130/JAK complex, activating both the JAK/STAT3 and MAPK (ERK1/2) pathways, thereby promoting osteoclast precursor differentiation and survival (68, 69). Similarly, CXCL8 interacts with CXCR1/CXCR2 receptors, triggering downstream PI3K–Akt and MAPK cascades, which synergize with NF-κB to enhance osteoclast maturation and the release of pro-angiogenic factors (70–72). These pathways not only promote osteoclastogenesis but also contribute to melanoma cell survival, invasiveness, and immune evasion by reshaping the bone metastatic niche (73, 74). Thus, cytokine-driven NF-κB and MAPK activation represents a crucial molecular bridge between immunosuppression and bone destruction in metastatic melanoma (75).

## 4 Melanoma-associated immunotherapy

### 4.1 Monotherapy with immune checkpoint inhibitors

**Figure 1:**
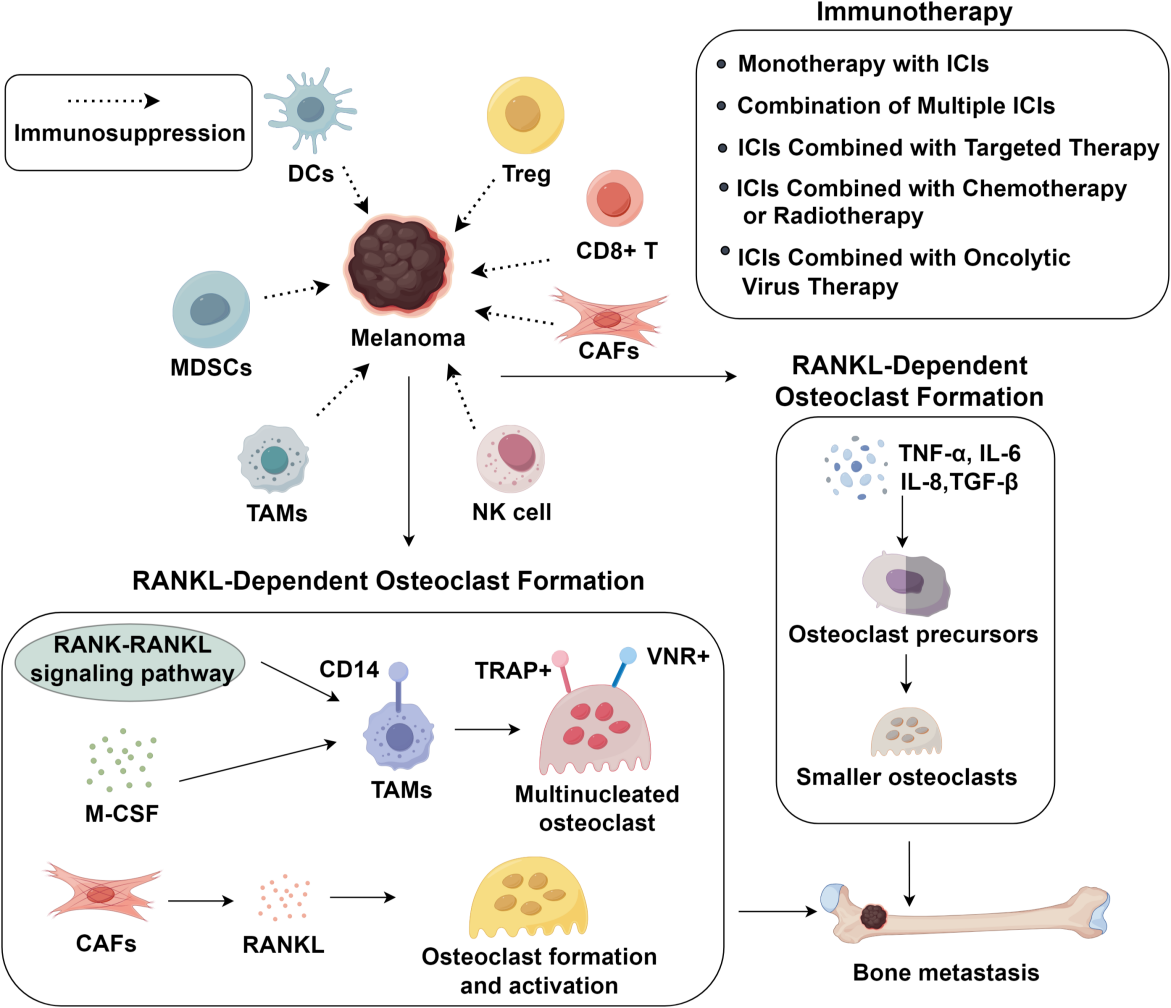
Immunosuppressive tumor microenvironment and advance in immunotherapy in melanoma bone metastasis.

CTLA-4, an inhibitory checkpoint receptor, suppresses T-cell activation by competing with CD28 for ligands CD80/CD86, depriving costimulatory signals (76). Ipilimumab, the first FDA-approved ICI, blocks CTLA-4-ligand interaction, enhancing tumor-infiltrating T-cells while suppressing Tregs in the TME (77). It significantly improves OS in metastatic melanoma versus chemotherapy, enabling durable disease control (11). PD-1/PD-L1 inhibitors are widely used ICIs. PD-1, expressed on T-cells, binds PD-L1 on tumor cells to inhibit T-cell function, facilitating immune evasion. Melanomas often overexpress PD-L1, correlating with poor prognosis. Anti-PD-1/PD-L1 antibodies (nivolumab, pembrolizumab, atezolizumab) disrupt this axis, enhancing antitumor immunity. Pembrolizumab shows superior response rates, PFS, and OS versus ipilimumab with reduced toxicity (78). Since 2017, anti-PD-1 monotherapy has served as adjuvant therapy for high-risk resected melanoma (50), though predictive biomarkers remain elusive. Despite these advances, the immunosuppressive TME frequently leads to primary or acquired ICI resistance (79). Mechanistically, melanoma cells upregulate alternative checkpoint receptors such as TIM−3, LAG−3, and TIGIT, which maintain T−cell exhaustion even after PD−1/PD−L1 or CTLA−4 blockade (80–82). In addition, activation of WNT/β−catenin signaling excludes dendritic cells and effector T cells from tumor lesions, generating a “cold” microenvironment that fails to respond to ICIs (83). Furthermore, metabolic suppressive pathways, including IDO1–mediated tryptophan depletion and arginase−1–driven arginine catabolism, diminish T−cell proliferation and effector function, further reinforcing immune evasion. Beyond PD-1/PD-L1 and CTLA-4, other checkpoints like TIM-3, TIGIT, and VISTA are co-expressed in melanoma TME, particularly on Tregs, marking them as potential targets (12) (**Figure 1**).

### 4.2 Combination therapies with ICIs

#### 4.2.1 Combination of multiple ICIs

Despite the survival benefits of ICI monotherapy, low response rates often require combination strategies. CTLA-4 and PD-1 inhibit T-cell activation via distinct mechanisms: CTLA-4 modulates early priming (lymph nodes), while PD-1 suppresses effector-phase proliferation (84). Their complementary actions suggest dual blockade may yield synergistic effects. A phase III trial demonstrated improved overall survival (OS) with ipilimumab-nivolumab combination versus monotherapy, but severe (grade 3/4) treatment-emergent adverse events (TEAEs) rose to 55.0% versus 27.3% with ipilimumab alone (85). This toxicity likely reflects systemic immune overactivation, necessitating optimization of dosing and sequencing to mitigate off-target effects. Beyond PD-1/CTLA-4 combinations, Opdualag (relatlimab-nivolumab) for unresectable/metastatic melanoma, targeting LAG-3, a next-generation checkpoint suppressing immunity via: (1) MHC II binding on antigen-presenting cells and (2) interaction with liver sinusoidal endothelial cell lectin on tumors, inhibiting CD4^+^/CD8^+^ T-cell function (86, 87). This innovation highlights the potential of novel dual-checkpoint strategies to broaden therapeutic efficacy while underscoring the need for improved safety profiles. Oncolytic virus (OV) therapy offers a distinct modality by selectively lysing tumor cells and initiating systemic immune activation. Upon intratumoral replication, OVs release tumor antigens and virions, triggering adaptive immunity against surrounding malignancies (88). The first OV for unresectable melanoma, has been shown to enhance T-cell infiltration and reverse PD-L1–mediated immunosuppression (88). Hence, combining OVs with ICIs may overcome drug resistance caused by high PD-L1 expression and restore antitumor immune responses (89).

#### 4.2.2 ICIs combined with chemotherapy or radiotherapy

Due to melanoma’s limited chemosensitivity, combining ICIs with chemotherapy is often used in advanced cases resistant to PD-1 blockade (90). Chemotherapeutics like dacarbazine, temozolomide, and platinum agents induce immunogenic cell death (ICD), releasing DAMPs and converting immunologically “cold” tumors into “hot” ones, enhancing ICI efficacy (88, 91). Preclinical studies show that ipilimumab combined with melphalan improves survival, reduces Tregs, and increases CD8^+^/Treg ratios in melanoma models (92). However, chemotherapy’s nonspecific cytotoxicity risks leukopenia, careful evaluation is warranted when combining ICIs and chemotherapy. Radiotherapy also exhibits immunomodulatory effects, promoting antigen presentation, type I interferon release, and a pro-inflammatory TME (93). Notably, radiotherapy induces the release of tumor-specific antigens, thus boosting T-cell–mediated tumor recognition (93). Clinical data indicate that radiotherapy and ICIs have synergistic effects. Saieg et al. (94) observed both local tumor regression and abscopal responses with ipilimumab-radiotherapy co-treatment. In unresectable or locally advanced melanoma, such combinations improved objective response and disease control without significantly increasing severe toxicity (95). For BRAFV600E-mutant melanoma, MAPK inhibitors achieve rapid responses, yet median progression-free survival remains under 12 months (96). In contrast, ICI offer more durable immunologic memory. Mechanistically, BRAF inhibition enhances tumor antigen presentation, synergizing with PD-1/PD-L1 blockade (97). The IMspire150 phase III trial confirmed that adding atezolizumab to vemurafenib and cobimetinib significantly improved median progression-free survival over dual-target therapy (98).

## 5 Conclusion

Melanoma’s aggressive progression and bone metastasis are orchestrated by a dynamic interplay between tumor cells and the immunosuppressive TME. Immunosuppressive cells including MDSCs, M2-polarized TAMs, Tregs, and CAFs drive immune evasion, osteoclast activation, and therapy resistance. While ICIs have transformed melanoma management, their efficacy is limited by the TME’s inhibitory landscape. Combination strategies, such as dual checkpoint blockade, ICI-targeted therapy, or oncolytic viruses, show promise in overcoming resistance by modulating immune cell function and enhancing antigen presentation. Future research should focus on identifying predictive biomarkers, optimizing therapeutic sequencing, and developing novel TME-targeted agents to improve durable responses. A deeper understanding of TME-immune crosstalk will be critical for advancing precision immunotherapy and mitigating skeletal complications in metastatic melanoma.
